# Epidemiology, species distribution, and outcome of nosocomial *Candida spp*. bloodstream infection in Shanghai: an 11-year retrospective analysis in a tertiary care hospital

**DOI:** 10.1186/s12941-021-00441-y

**Published:** 2021-05-13

**Authors:** Yan-Jun Zheng, Ting Xie, Lin Wu, Xiao-Ying Liu, Ling Zhu, Ying Chen, En-Qiang Mao, Li-Zhong Han, Er-Zhen Chen, Zhi-Tao Yang

**Affiliations:** 1 Department of Emergency, Ruijin Hospital, Shanghai Jiao Tong University School of Medicine, Shanghai, 200025 China; 2 Emergency Center, Suining Central Hospital, Suining, 629000 Sichuan Province China; 3 Department of Geriatrics, Ruijin Hospital, Shanghai Jiao Tong University School of Medicine, Shanghai, China; 4 Department of Cardiovascular Surgery, Ruijin Hospital, Shanghai Jiao Tong University School of Medicine, Shanghai, China; 5 Department of Emergency, Ruijin North Hospital, Shanghai Jiao Tong University School of Medicine, Shanghai, 201801 China; 6 Department of Laboratory Medicine, Ruijin Hospital, Shanghai Jiao Tong University School of Medicine, Shanghai, China; 7 Pôle Sino-Français de Recherches en Science du Vivant Et Génomique, Ruijin Hospital, Shanghai Jiao Tong University School of Medicine, Shanghai, China

**Keywords:** *Candida spp.*, Bloodstream infection, Epidemiology, Species distribution, Antifungal therapy, Early treatment

## Abstract

**Background:**

The incidence of *Candida* bloodstream infections (BSIs), has increased over time. In this study, we aimed to describe the current epidemiology of *Candida* BSI in a large tertiary care hospital in Shanghai and to determine the risk factors of 28-day mortality and the impact of antifungal therapy on clinical outcomes.

**Methods:**

All consecutive adult inpatients with *Candida* BSI at Ruijin Hospital between January 1, 2008, and December 31, 2018, were enrolled. Underlying diseases, clinical severity, species distribution, antifungal therapy, and their impact on the outcomes were analyzed.

**Results:**

Among the 370 inpatients with 393 consecutive episodes of *Candida* BSI, the incidence of nosocomial *Candida* BSI was 0.39 episodes/1000 hospitalized patients. Of the 393 cases, 299 (76.1%) were treated with antifungal therapy (247 and 52 were treated with early appropriate and targeted antifungal therapy, respectively). The overall 28-day mortality rate was 28.5%, which was significantly lower in those who received early appropriate (25.5%) or targeted (23.1%) antifungal therapy than in those who did not (39.4%; P = 0.012 and P = 0.046, respectively). In multivariate Cox regression analysis, age, chronic renal failure, mechanical ventilation, and severe neutropenia were found to be independent risk factors of the 28-day mortality rate. Patients who received antifungal therapy had a lower mortality risk than did those who did not.

**Conclusions:**

The incidence of *Candida* BSI has increased steadily in the past 11 years at our tertiary care hospital in Shanghai. Antifungal therapy influenced short-term survival, but no significant difference in mortality was observed between patients who received early appropriate and targeted antifungal therapy.

## Background

The incidence of invasive fungal infection has increased over time, especially for *Candida* bloodstream infections (BSIs), which is associated with considerable excess mortality and costs [1–3]. In the past two decades, the incidence of fungal infection has increased from 0.1 episodes/1000 admissions to 0.3–0.6 episodes/1000 admissions in China, North America, and several European countries [4–7]. The *candida* BSI mortality rate ranges from 35 to 53% [8–11]. The optimal management of *Candida* BSIs includes early awareness of patients at risk, control of the infection source, and timely administration of appropriate antifungal agents [12, 13]. Consequently, antifungal agents have been widely used as empirical therapy. However, the overuse of antifungal agents results in increased costs, toxicity, and ecological selection pressure for antifungal resistance and adverse drug interactions. Several studies showed that delayed antifungal therapy (more than 48 h from onset) is associated with higher mortality [14], whereas others have reported conflicting results [15–17].

In this study, we retrospectively analyzed data from all patients with *Candida* BSI at our hospital between 2008 and 2018, aiming to describe their clinical characteristics, species distribution, antifungal therapy and to determine the risk factors for 28-day mortality.

## Methods

### Study setting and population

A retrospective analysis of data on consecutive *Candida* BSI episodes in adult inpatients (≥ 18 years) between January 1, 2008, and December 31, 2018, collected from the microbiology database of a 1900-bed teaching hospital in Shanghai, was performed.

Demographics and data on underlying diseases, comorbidities, severity of clinical features, *Candida* species distribution, and early appropriate or targeted antifungal treatment were compared among the patients with *Candida* BSI. Data on the initial and targeted antifungal agents used were also collected.

For each patient, aerobic and anaerobic blood culture bottles were each inoculated with 10 ml of blood and transported to the laboratory within 1 h of collection. An episode of candidemia was defined as the first isolation (incident candidemia) of *Candida* species from blood culture in a patient with signs of infection. Blood cultures of patients with long lines (central venous catheter (CVC), Hickman or PICC) were taken through both peripheral vein and longlines, instances where longlines blood culture were positive but the peripheral blood culture remained negative indicate colonization of the catheter rather than BSI. New positive blood cultures within 30 days from the incident candidemia were considered part of the same episode [18]. BSI was considered as nosocomial if diagnosed at least 48 h after hospital admission [19, 20]. We evaluated the clinical manifestations and host factors when confirming whether it was genuine candidemia. Severe neutropenia was defined as < 500/mm^3^ absolute neutrophil count. Prior corticosteroid was defined as receiving > 1 mg/kg/d prednisone for more than one week or equivalent before *Candida* BSI onset.

### Laboratory methods

Isolates were detected from blood cultures using the BACTEC™ FX system (Becton Dickinson, Inc., Sparks, MD, USA), identified using VITEK-2 system (bioMérieux, Marcy-l’Étoile, France) before 2015, and matrix-assisted laser desorption ionization-time of flight mass spectrometer (bioMérieux, Marcy-l’Étoile, France) after 2015. Susceptibility testing for flucytosine, amphotericin B, fluconazole, voriconazole, and itraconazole was performed using the ATB® FUNGUS 3 system (BioMérieux, France), which is widely used in China [21]. This test provides information on susceptibility to antifungals agents, which is concordant with that obtained using the methodologies of the Clinical and Laboratory Standards Institute (CLSI) and European Committee on Antimicrobial Susceptibility Testing (EUCAST) [22].

Early appropriate antifungal treatment was defined as commencement of appropriate drug treatment at an adequate dosage before obtaining in vitro susceptibility test results. The adequate dosage of the antifungal agent was defined according to 2009 or 2016 Infectious Diseases Society of America (IDSA) guidelines [18, 23]. Targeted antifungal treatment was defined as commencement of appropriate targeted treatment after obtaining results from susceptibility testing, regardless of whether the antifungal treatment initiated was appropriate. Crude mortality was calculated from data on deaths registered 28 days after the occurrence of *Candida* BSI.

### Statistical analysis

Descriptive and subgroup analyses were performed for the baseline characteristics, and continuous variables were expressed as mean ± standard deviation (SD) or median and interquartile range according to their distributions. The chi-square test or 2-tailed Fisher exact test was applied to categorical variables. To identify the risk factors for 28-day mortality, multivariate Cox regression analysis was performed, and adjusted hazard ratio (HRs) with 95% confidence intervals (CIs) were reported. Variables that were associated with 28-day mortality in the Cox univariate analyses with a P < 0.05 were entered into the multivariate Cox regression analysis model based on the forward selection. Two-tailed tests of significance at the level of a P value < 0.05 level was considered as significant. Statistical analysis was performed using IBM SPSS Statistics for Windows, version 22.0 (IBM Corp., Armonk, N.Y. USA).

### Ethics

The study was approved by Ruijin Hospital, Shanghai Jiao Tong University, School of Medicine institutional review board, and written informed consent was not required because of the observational nature of this study.

## Results

### Incidence and clinical features of *Candida* BSI episodes

**Fig. 1:**
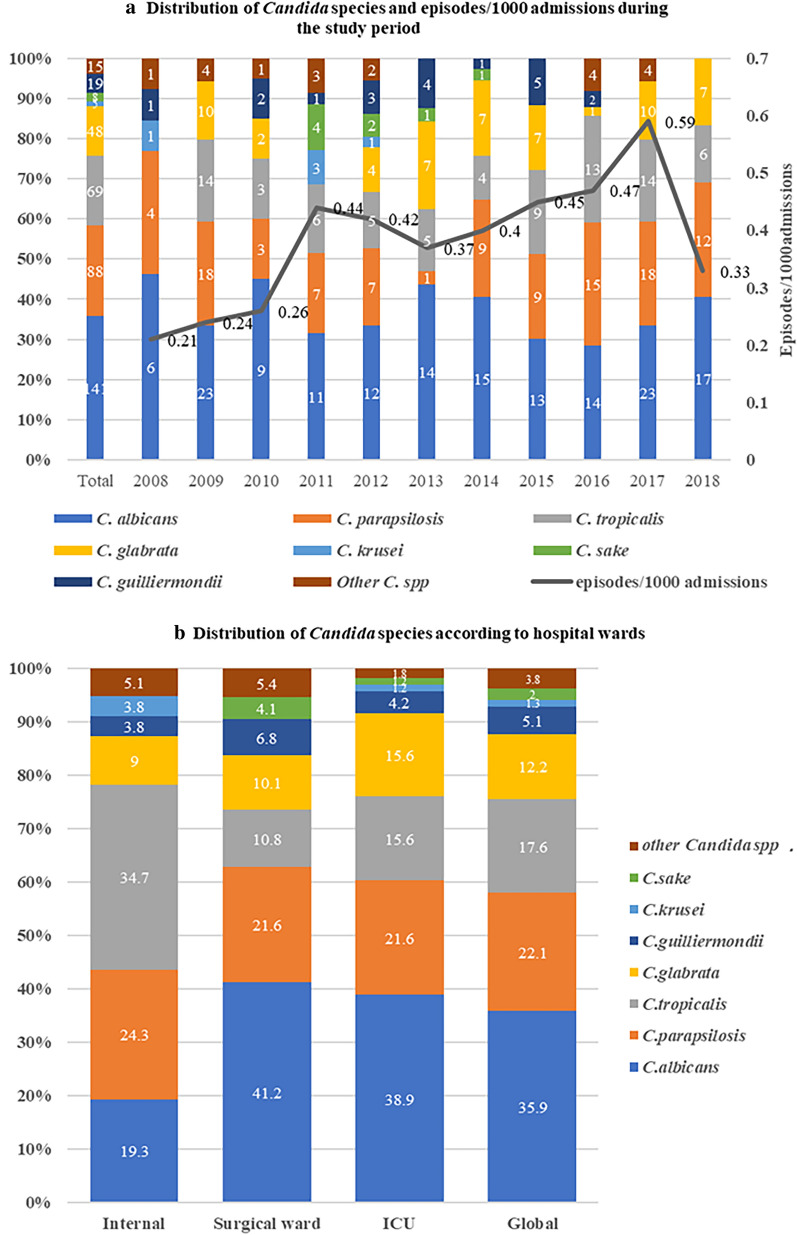
**a** Distribution of *Candida spp.* and episodes/1000 admissions during the study period. **b** Distribution of *Candida spp.* according to hospital wards;148 episodes were from surgical wards; 167 episodes were from ICUs and 78 episodes were from the internal medicine wards

Data on a total of 393 consecutive episodes of *Candida* BSI were collected from 370 inpatients during the 11-year study period. No outbreaks were reported during this period. The demographic characteristics of the patients are summarized in Table 1. The mean age of the patients was 57.6 ± 19.0 years, and 74.3% were male. *Candida* BSI incidence was 0.39 episodes/1000 admissions. The incidence increased steadily from 0.21 (2008), to 0.59 (2017), to 0.33 episodes per 1,000 admissions (2018) (Fig. 1a). Among the 393 *Candida* BSI episodes, 148 (37.7%), 167 (42.5%), and 78 (19.8%) occurred in the surgical ward, intensive care units (ICUs), and internal medicine ward, respectively (Fig. 1b).

**Table 1:** Demographic and clinical data for patients with *Candida* bloodstream infection

	*C. albicans*	*C. parapsilosis*	*C. tropicalis*	*C. glabrata*	*C. guilliermondii*	*C* *sake*	*C* *krusei*	Other *Candida spp.*	Total
	(n = 141)	(n = 87)	(n = 69)	(n = 48)	(n = 20)	(n = 8)	(n = 5)	(n = 15)	(n = 393)
Age, years	65.2 ± 14.5	53.2 ± 20.3	50.5 ± 19.5	60.7 ± 17.5	50.5 ± 19.4	52 ± 20.6	40.8 ± 27.1	52.6 ± 20.0	57.6 ± 19.0
Male	102(73.4)	60(69.0)	51(73.9)	44(91.7)	14(70)	8(100)	2(40)	11(73.3)	292(74.3)
Origin									
Internal medicine ward	15(10.6)	19(21.8)	27(39.1)	7(14.6)	3(15)	0(0)	3(60)	4(26.7)	78(19.8)
Surgical ward	61(43.3)	32(36.8)	16(23.2)	15(31.3)	10(50)	6(75)	0(0)	8(53.3)	148(37.7)
ICU	65(46.1)	36(41.4)	26(37.7)	26(54.1)	7(35)	2(25)	2(40)	3(20)	167(42.5)
Time from admission to infection, days	30.6 ± 35.3	48.4 ± 56.2	37.7 ± 32.4	27.5 ± 19.1	35.9 ± 52.5	120.6 ± 242.7	64.2 ± 68.6	21.5 ± 13.6	37.6 ± 53.1
Length of hospital stay, days	56.0 ± 54.9	83.2 ± 78.1	71.5 ± 56.1	67.1 ± 72.7	97.7 ± 142.6	178.5 ± 281.7	92.4 ± 88.5	52.7 ± 101.5	71.1 ± 82.8
Turnaround time, days	4.3 ± 1.9	4.5 ± 1.1	3.8 ± 1.1	4.8 ± 1.3	4.3 ± 1.2	4.5 ± 1.6	4.4 ± 1.5	5.9 ± 1.8	4.4 ± 1.5
Underlying disease									
Solid tumor	47(33.3)	26(29.9)	13(18.8)	17(35.4)	6(30)	2(25)	0(0)	7(46.7)	118(30)
Hematologic malignancy	8(5.7)	6(6.9)	24(34.8)	2(4.2)	2(10)	1(12.5)	3(60)	2(13.3)	48(12.2)
Diabetes mellitus	33(23.4)	21(24.1)	8(11.6)	9(18.8)	1(5)	1(12.5)	0(0)	4(26.7)	77(19.6)
Chronic cardiac disease	55(39)	22(25.3)	16(23.2)	18(37.5)	6(30)	3(37.5)	2(40)	2(13.3)	124(31.6)
Chronic pulmonary disease	26(18.4)	9(10.3)	5(7.2)	6(12.5)	1(5)	1(12.5)	1(20)	3(20)	52(13.2)
Chronic renal failure	18(12.8)	6(6.9)	6(8.7)	5(10.4)	5(25)	0(0)	0(0)	2(13.3)	42(10.7)
Skin barrier compromised	5(3.5)	9(10.3)	5(7.2)	1(2.1)	3(15)	3(37.5)	0(0)	0(0)	26(6.6)
Prior surgical intervention (< 1 month)	97(68.8)	48(55.2)	36(52.2)	30(62.5)	15(75)	6(75)	2(40)	10(66.7)	244(62.1)
Corticosteroid use	11(7.8)	13(14.9)	12(17.4)	9(18.8)	4(20)	0(0)	3(60)	2(13.3)	54(13.7)
Prior use of antifungal agents (< 6 months)	20(14.2)	19(21.8)	24(34.8)	11(22.9)	8(40)	1(12.5)	4(80)	1(6.7)	88(22.4)
Severity of clinical feature									
Fever (T > 38.2 ℃)	114(80.9)	63(72.4)	60(87)	34(70.8)	16(80)	6(75)	3(60)	13(86.7)	309(78.6)
Parenteral nutrition	71(50.4)	42(48.3)	28(40.6)	23(47.9)	5(25)	3(37.5)	2(40)	6(40)	180(45.8)
Mechanical ventilation	58(41.1)	31(35.6)	23(33.3)	25(52.1)	5(25)	2(25)	2(40)	1(6.7)	147(37.4)
Renal replacement therapy	17(12.1)	10(11.5)	9(13)	7(14.6)	5(25)	0(0)	1(20)	0(0)	49(12.5)
Central venous catheter	121(85.8)	67(77)	49(71)	43(89.6)	17(85)	5(62.5)	4(80)	10(66.7)	316(80.4)
Severe neutropenia	4(2.8)	8(9.2)	22(31.9)	1(2.1)	2(10)	0(0)	3(60)	2(13.3)	42(10.7)
28-day mortality	54(38.3)	16(18.4)	19(27.5)	13(27.1)	3(15)	1(12.5)	1(20)	5(33.3)	112(28.5)

Other *Candida spp.* Includes *C. gum* (4 cases), *C. lusitaniae* (3 cases), *C. intermedia* (2 cases), *C. lipolytica* (2cases), *C. theae* (2 cases), *C.famata* (1case), and *C. haemulonii* (1 case)

Data were expressed as mean ± SD for continuous variables and n (%) for categorical variables

*ICU* intensive care unit, *SD* standard deviation

*C. albicans* was isolated in 19.3, 41.2, and 38.9%, of cases in internal medicine wards, surgery wards, and the ICU, respectively (P = 0.003). A higher proportion of *C. tropicalis* (34.7%) was found in internal medicine wards than in the surgery wards (21.6%) and ICUs (21.6%).

Most patients with *Candida* BSI had at least one comorbidity. These included 118 (30%) patients with solid tumors, 48 (12.2%) had hematological malignancies, 77 (19.6%) had diabetes mellitus, 124 (31.6%) had chronic cardiac disease, 52 (13.2%) had chronic pulmonary disease, 42 (10.7%) had chronic renal failure, in 26 (6.6%) patients, the skin barrier was considered compromised, 244 (62.1%) had prior surgical intervention, 54 (13.7%) used corticosteroid, 88 (22.4%) used prior antifungal agents, and 255 (64.9%) received antibiotics prior *Candida* BSI onset. A total of 244 (72%) patients had at least two comorbidities. Regarding the severity, 309 (78.6%) patients had fever, 180 (45.8%) received parenteral nutrition, 147 (37.4%) received mechanical ventilation, 49 (12.5%) received renal replacement therapy, and 42 (10.7%) had severe neutropenia. The clinical characteristics of patients by *Candida* species are shown in Table 1.

### Antifungal susceptibility of *Candida* isolates

A total of 393 *Candida* spp. were isolated, including 141 (35.9%), *C. albicans,* 87, *C. parapsilosis* (22.1%); 69, *C. tropicalis* (17.6%); 48, *C. glabrata* (12.2%); 20, *C. guilliermondii* (5.1%); 8, *C. sake* (2.0%); 5, *C.krusei* (1.3%); and 15, other species (4, *C. gum*; 3, *C. lusitaniae*; 2, *C. intermedia*; 2, *C. theae*; 2, *C. lipolytica*; 1, *C. famata*; and 1, *C. haemulonii*).

Among the 393 *Candida* species, 378 were subjected to antifungal susceptibility testing, based on 2012 CLSI breakpoints (CBPs) [24]. As shown in Table 2, the susceptibility of *C. albicans, C. parapsilosis* to fluconazole, and voriconazole were quite high, compared to that of itraconazole (94, 93.3 vs. 82.1%). The susceptibility of *C. tropicalis* to triazoles fluconazole, voriconazole, and itraconazole was unsatisfactory. Amphotericin B and 5-flucytosine remained superior against common *Candida spp.*, except for *C. krusei* and *C. guilliermondii*, with 95% susceptibility.

**Table 2:** Antifungal susceptibility testing results (ATB Fungus 3) of 378 *Candida* [n (%)]

	*C.albicans* (n = 134)	*C.parapsilosis* (n = 86)	*C.tropicalis* (n = 67)	*C.glabrata* (n = 47)	*C.krusei* (n = 5)	*C.sake* (n = 8)	*C.guilliermondii* (n = 19)	Other *Candida spp.* (n = 12)	Total(n = 378)
Fluconazole									
S	126 (94)	77(89.5)	35 (52.2)	0 (0)	0 (0)	8(100)	13 (68.4)	9 (75.0)	268 (70.9)
SDD	1 (0.8)	6 (7.0)	3 (4.5)	44(93.6)	0 (0)	0 (0)	0 (0)	0 (0)	54 (14.3)
R	7 (5.2)	3 (3.5)	29 (43.3)	3 (6.4)	5 (100)	0 (0)	6 (31.6)	3 (25.0)	56 (14.8)
Itraconazole									
S	110 (82.1)	75 (87.2)	25 (37.3)	0 (0)	0 (0)	8 (100)	6 (31.6)	9 (75.0)	233 (61.6)
SDD	6 (4.5)	7 (8.1)	4 (6.0)	40 (85.1)	2 (40.0)	0 (0)	7 (36.8)	0 (0)	66 (17.5)
R	18 (13.4)	4 (4.7)	38 (56.7)	7 (14.9)	3 (60.0)	0 (0)	6 (31.6)	3 (25.0)	79 (20.9)
Voriconazole									
S	125 (93.3)	79 (91.9)	41 (61.2)	45 (95.8)	4 (80.0)	8 (100)	12 (63.2)	11 (91.7)	325 (86.0)
SDD	0 (0)	2 (2.3)	2 (3.0)	1 (2.1)	1 (2.0)	0 (0)	3 (15.8)	0 (0)	9 (2.4)
R	9 (6.7)	5 (5.8)	24 (35.8)	1 (2.1)	0 (0)	0 (0)	4 (21.0)	1 (8.3)	44 (11.6)
Amphotericin B									
S	133 (99.3)	83 (96.5)	67 (100)	47 (100)	5 (100)	8 (100)	18 (94.7)	11 (91.7)	372 (98.4)
R	1 (0.7)	3 (3.5)	0 (0)	0 (0)	0 (0)	0 (0)	1 (5.3)	1 (8.3)	6 (1.6)
Flucytosine									
S	132 (98.5)	85 (98.8)	65 (97.0)	46 (97.9)	1 (20.0)	8 (100)	9 (47.4)	12 (100)	358 (94.7)
R	2 (1.5)	1 (1.2)	2 (3.0)	1 (2.1)	4 (80.0)	0 (0)	10 (52.6)	0 (0)	20 (5.3)

15 *Candida spp.* isolates did not have a susceptibility test, *C. albicans* (7), C. *parapsilosis* (2), *C. tropicalis* (2), and *glabrata*, *theae*, *gum*, *haemulonii* each

*R* resistance, *S* susceptible, *SDD* susceptible dose dependence

### Antifungal therapy and outcome of patients with *Candida* BSI

Antifungal therapy was administered to 299 (76.1%) patients, whereas 94 (23.9%) patients did not receive antifungal therapy. The Comparison between the patients receiving antifungal therapy and those without antifungal therapy was shown in Table 3. Among those who received antifungal therapy, 247 (62.8%) received early appropriate antifungal therapy, and 52 (13.2%) received targeted antifungal therapy. Fluconazole was most frequently used as empirical therapy, followed by echinocandins and voriconazole. Eighteen (4.6%) patients with *Candida* BSI received combination therapy.

**Table 3:** Comparison between the patients receiving antifungal therapy and without antifungal therapy

	Antifungal therapy(n = 299)	No antifungal treatment(n = 94)	P value
Age	56.47 ± 19.19	61.13 ± 17.81	0.038
Male	223(74.6)	69(73.4)	0.820
Underlying disease			
Solid tumor	80(26.8)	38(40.4)	0.012
Hematologic malignancy	39(13.0)	9(9.6)	0.370
Diabetes mellitus	56(18.7)	21(22.3)	0.442
Chronic Cardiac disease	88(29.4)	36(38.3)	0.107
Chronic Pulmonary disease	44(14.7)	8(8.5)	0.121
Chronic renal failure	29(9.7)	13(13.8)	0.258
Skin barrier compromised	21(7.0)	5(5.3)	0.016
Prior surgical intervention (< 1 month)	182(60.9)	62(66.0)	0.375
Corticosteroid use	45(15.1)	9(9.6)	0.179
Prior antifungal agents use (< 6 month)	76(25.4)	12(12.8)	0.010
Severity of clinical feature			
Fever (T > 38.2℃)	244(81.6)	65(69.1)	0.010
Parenteral nutrition	135(45.2)	45(47.9)	0.644
Mechanical ventilation	113(37.8)	34(36.2)	0.777
Renal replacement therapy	36(12.0)	13(13.8)	0.647
Central venous catheter	242(80.9)	74(78.7)	0.637
Severe neutropenia	36(12.0)	6(6.4)	0.338
APACHE II score	11.86 ± 5.91	11.72 ± 5.37	0.928
SOFA score	3.66 ± 3.56	4.22 ± 4.24	0.577

**Fig.2:**
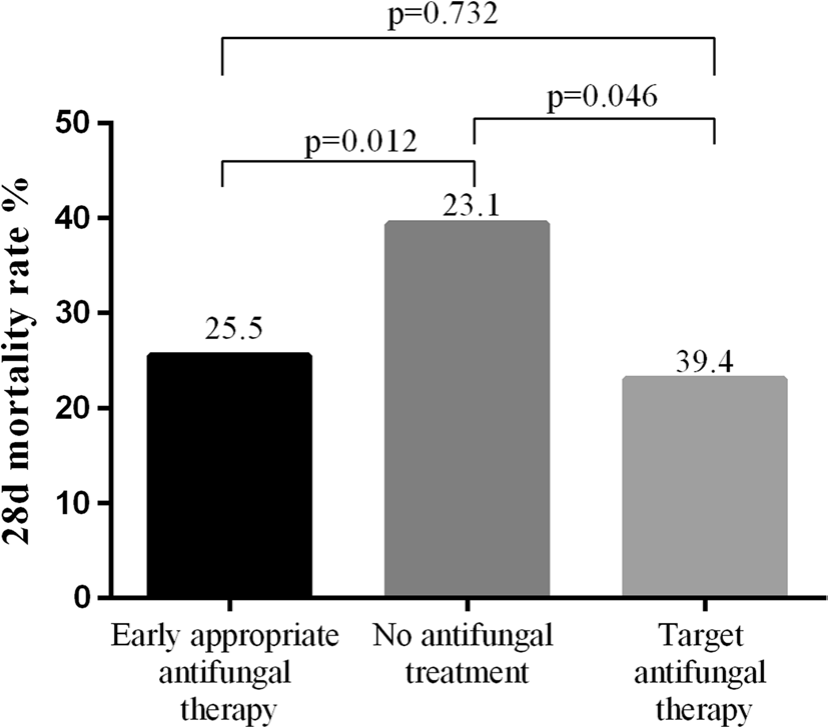
Relationship between hospital mortality (28-day) and the timing of antifungal treatment

The overall, 28-day mortality rate was 28.5%, and the rate was significantly higher in internal medicine wards and ICUs than in surgical wards (37.2% and 34.7% vs. 16.9%, respectively, P < 0.001). As shown in Fig. 2, the mortality rates among those who received early appropriate or targeted antifungal therapy were 26.8% and 25.1% (P = 0.012 or P = 0.046), compared to 39.3% for those who did not receive any antifungal therapy, with no significant difference (P = 0.732) between those who received early appropriate antifungal therapy and those who received targeted antifungal therapy.

On univariate analysis, age, solid tumor, diabetes mellitus, chronic cardiac disease, chronic renal failure, skin disease, prior surgical intervention, mechanical ventilation, severe neutropenia, and antifungal therapy were found to be associated with 28-day mortality. On multivariate Cox regression analysis, advanced age (HR = 1.025; 95%CI, 1.013–1.037; P < 0.001), chronic renal failure (HR = 2.018; 95%CI 1.234–3.299; P = 0.005), mechanical ventilation (HR = 1.950; 95%CI 1.307–2.912; P = 0.001), and severe neutropenia (HR = 4.347; 95%CI 2.462–7.675; P < 0.001), were found to be independent risk factors for 28-day mortality. However, antifungal therapy (HR = 0.570; 95%CI 0.382–0.849; P = 0.006) was an independent protective factor for 28-day mortality (Table 4).

**Table 4:** Multivariable Cox regression analysis for 393 *Candida* bloodstream infection episodes

	Survival (n = 281)	28-day outcome		Multivariable analysis		
		Death (n = 112)	P-value	HR (95%CI)	P-value	
Male	216(76.9)	76(67.9)	0.065	-	-	
Age, years	55.2(19.5)	63.6(16.2)	< 0.01	1.025(1.013–1.037)	< 0.001	
Underlying disease				-	-	
Solid tumor	91(32.4)	27(24.1)	0.106	-	-	
Hematologic malignancy	32(11.4)	16(14.3)	0.428	-	-	
Diabetes mellitus	49(17.4)	28(25)	0.088	-	-	
Chronic Cardiac disease	72(25.6)	52(46.4)	< 0.01	-	0.105	
Chronic Pulmonary disease	34(12.1)	18(16.1)	0.294	-	-	
Chronic renal failure	20(7.1)	22(19.6)	< 0.01	2.018(1.234–3.299)	0.005	
Skin barrier compromised	24(8.5)	2(1.8)	0.015	-	0.308	
Prior surgical intervention (< 1 month)	182(64.8)	62(55.4)	0.083	-	-	
Corticosteroid use	40(14.2)	14(12.5)	0.652	-	-	
Prior antifungal agents use (< 6 month)	64(22.8)	24(21.4)	0.772	-	-	
Severity of clinical feature				-	-	
Fever (T > 38.2℃)	220(78.3)	89(79.5)	0.798	-	-	
Parenteral nutrition	124(44.1)	56(50)	0.292	-	-	
Mechanical ventilation	89(31.7)	58(51.8)	< 0.01	1.950(1.307–2.912)	0.001	
Renal replacement therapy	32(11.4)	17(15.2)	0.305	-	-	
Central venous catheter	227(80.8)	89(79.5)	0.766	-	-	
Severe neutropenia	24(8.5)	18(16.1)	0.029	4.347(2.462–7.675)	< 0.001	
Antifungal therapy	224(74.9)	75(25.1)	0.007	0.502(0.294–0.857)	0.006	
No treatment	57(60.6)	37(39.4)				

Data were expressed as mean ± SD for continuous variables and n (%) for categorical variables

*SD* standard deviation

## Discussion

Our study showed that the incidence of *Candida* BSI has increased steadily in the past 11 years at our tertiary care hospital in Shanghai. Several studies have shown a substantial increase in *Candida* BSI incidence in the past two decades, which is similar to our study findings [4, 11, 25].

*C. albicans* remains the most common pathogen causing *Candida* BSI. However, over the past two decades, an increased percentage of common non-C. *albicans Candida spp.* have been reported worldwide. In our study, non-C. *albicans* accounted for 64.1%. C. *parapsilosis*, C. *tropicalis,* and C. *glabrata* accounted for most of the non-C. *albicans* species. However, some studies in western countries showed that C. *glabrata* was the most frequent species among non-C. *albicans*, whereas C. *tropicalis* was quite rare compared to the incidence reported in our study (5.9 and 7.0 vs. 27.4%) [26, 27]. In this study, the incidence of C. *guilliermondii* was significantly higher than that in other studies (4.8 vs*.* 0.4%) [28].

The antifungal susceptibility testing showed that the susceptibility of C. *albicans*,

C. *parapsilosis* and C. *sake* to fluconazole were quite high (94, 89.5, 100%). On the other hand, the resistance rate of C. *tropicalis* for fluconazole was as high as 43.3%, which was significantly higher than those reported in other studies abroad [29, 30]. This may deserve more attention from clinicians in actual application. However, the antifungal results in our study were according to 2012 CLSI, since we didn’t have the MIC values for the antifungals, we couldn’t reanalyze according to new CLSI guidelines on antifungal susceptibility M60 published in 2017. The susceptibility testing in our study was performed using the ATB® FUNGUS 3 system, there were some contradictory views about its performance. Zhang L et al. thought ATB® FUNGUS 3 might misleading high MICs of *Candida* spp. to azoles[31], while some other studies support ATB® FUNGUS system was an objective, reproducible and simple method for the accurate determination of MICs of the most common antifungal drugs in yeasts[32, 33].

In our hospital, the physicians administrated prophylactic antifungal therapy and empirical therapy according to the patient's risk factors and clinical manifestation. Fluconazole (800-mg loading dose, then 400 mg daily) was the most used prophylactic antifungal agent during the study period. Fluconazole (800-mg loading dose, then 400 mg daily) and caspofungin (loading dose of 70 mg, then 50 mg daily) were the most used empirical therapies[18], after IDSA 2016 guidelines published[23], caspofungin was preferred over fluconazole. Amphotericin B and voriconazole were more used as target therapies.

The contributing factors of the finding of quite a low 28-day mortality rate for *Candida* BSI in patients from surgical wards compared to those of other wards need further study.

Since there was no significant difference in the 28-day mortality rate between the patients who received early appropriate (26.8%) or targeted antifungal therapy (25.1%), we further analyzed the demographic data, underlying diseases, and clinical features between these two groups. We found no significant differences between the two groups in age (P = 0.33), sex (P = 0.89), number of underlying diseases (P = 0.32), number of severe clinical features (P= 0.96), APACHE II score (P= 0.072), or SOFA score(P= 0.310). The choice of antifungal agents between these two groups was further analyzed. In the early appropriate antifungal therapy group, the rate of azoles use was 50.6%, and the rate of echinocandin use was 30.3%. In the targeted antifungal therapy group, the rate of azoles use was 65.4%, and the rate of echinocandin use was 17.3%. The rate of echinocandin use was lower in the targeted antifungal therapy group than in the early appropriate antifungal therapy group (P= 0.038). Is the lower rate of echinocandin use in the targeted antifungal therapy group one of the reasons that caused no significant difference in mortality between the two groups? Many studies have confirmed the important role of echinocandin in antifungal therapy. Echinocandin has been recommended as a first-line antifungal agent since 2009[18], with fluconazole as an acceptable alternative for selected patients, reflecting the efficacy demonstrated by echinocandins and increasing resistance observed with fluconazole[34, 35]. Therefore, we believe that a lower rate of echinocandin use in the delayed antifungal treatment group is unlikely to lead to a decrease in the 28-day mortality rate.

Based on the above analysis, we suggest that early antifungal therapy has no significant impact on the 28-day mortality rate compared with targeted antifungal therapy. Many clinicians currently administer empirical antifungal agents, and our study results could be used to guide the clinical care of their patients. Overuse of antifungal drugs inevitably leads to a waste of medical resources and increased drug resistance [36]. Several studies support our findings [15–17] that the severity of illness (APACHE-II Score) affected short-term survival in patients with *Candida* infection, whereas the choice of initial antifungal agents did not affect short-term survival. Trifi et al. revealed no beneficial impact of an empirical antifungal therapy on 28- day survival or in preventing the occurrence of candidemia in critically ill patients with non-neutropenic sepsis [37]. However, some studies differed in their findings on the timing of antifungal agents' use. Bassetti et al. showed that the use of antifungal agents within 48 h of obtaining a positive blood culture result is an independent protective factor against mortality during hospitalization [14]. Similarly, Tedeschi et al. showed that the administration of appropriate antifungal agents within 72 h after a positive blood culture is a protective factor for mortality during hospitalization [38]. Our results revealed similar risk factors (age, severe neutropenia, and mechanical ventilation) and protective factors (antifungal therapy) for 28-day mortality.

Although our data were collected from a large hospital in Shanghai, several limitations of this study should be taken into consideration. First, this was a retrospective study performed at a single center, which could lead to selection bias. Also, the antifungal results in our study were according to 2012 CLSI, rather than the 2017 CLSI guidelines on antifungal susceptibility M60. Further, Echinocandin susceptibility testing has not been carried out at our hospital, relevant clinical data could not be obtained. Moreover, the timing of CVC removal, laboratory tests such as PCT, CRP, and (1, 3)-β-D-glucan were not included for analysis because of missing data.

## Conclusion

Our retrospective study findings showed an increased incidence of *Candida* BSI in the past 11 years in Shanghai. Although the percentage of non- C. *albicans* spp. has been increasing, C. *albicans* spp. remains the most frequently isolated species. The mortality of patients with *Candida* BSI was quite high (28.5%), especially in internal medicine wards (37.2%). Antifungal therapy improved the short-term survival of patients with *Candida* BSI. Whether preemptive antifungal therapy should be initiated or antifungal therapy should be initiated after the antifungal susceptibility test needs further discussion.

## Data Availability

All data generated or analyzed during this study are included in this published article.

## Abbreviations

BSI: Bloodstream infection
CI: Confidence interval
CLSI: Clinical and Laboratory Standards Institute
CVC: Central venous catheter
EUCAST: European Committee on Antimicrobial Susceptibility Testing
HR: Hazard ratio
HIV: Human immunodeficiency virus
ICU: Intensive care unit
IDSA: Infectious Diseases Society of America
SD: Standard deviation
